# Limitations of MELD in Isolated Polycystic Liver Disease: Fatal Traumatic Cyst Rupture Complicated by Abdominal Compartment Syndrome

**DOI:** 10.1155/crhe/5849774

**Published:** 2026-08-16

**Authors:** Zachary C. Vinton, Carter A. Schulz, Stephanie J. Melquist, Clark C. Kulig

**Affiliations:** ^1^ Department of Internal Medicine, University of Nebraska Medical Center, Omaha, Nebraska, USA, unmc.edu; ^2^ Department of Gastroenterology, University of Michigan Health, Ann Arbor, Michigan, USA, uofmhealth.org; ^3^ Department of Transplant Hepatology, AdventHealth Porter, Denver, Colorado, USA

## Abstract

Isolated polycystic liver disease (PLD) is rare and may cause debilitating mass‐effect symptoms despite relatively preserved hepatic synthetic function. Because the Model for End‐Stage Liver Disease (MELD) score does not capture nutritional, functional, and mechanical disease burden, affected patients may have low calculated MELD scores despite advanced symptomatic disease. Updated MELD exception guidance broadened consideration for PLD to include malnutrition and sarcopenia. We describe a patient with isolated PLD who was initially considered too well for transplantation but subsequently developed progressive weight loss, sarcopenia, and functional decline, prompting repeat evaluation, wait‐list registration, and MELD exception approval. Several weeks after listing, he sustained traumatic hepatic cyst rupture, resulting in fatal hemoperitoneum and abdominal compartment syndrome. This case highlights the evolving clinical course of symptomatic PLD, the importance of longitudinal reassessment, and the potential severity of rare cyst‐related complications.

## 1. Introduction

Polycystic liver disease (PLD) most commonly occurs in association with autosomal dominant polycystic kidney disease (ADPKD), which has a prevalence of approximately 1:400–1:1000 [1]. Up to 80%–90% of patients with PLD also have ADPKD [2]. In contrast, isolated PLD—defined as diffuse hepatic cystic disease in the absence of renal involvement—is far less common, with an estimated prevalence of 1:100,000–1:1,000,000 [1]. Most individuals with isolated PLD remain asymptomatic and are diagnosed incidentally on imaging; however, extensive cyst burden can result in progressive hepatomegaly, portal hypertension, and, in advanced cases, severe symptomatic liver disease requiring transplantation [3, 4].

Although uncommon complications such as cyst hemorrhage or rupture have been described, severe outcomes such as massive hemoperitoneum with hemorrhagic shock are exceedingly rare [5]. To our knowledge, abdominal compartment syndrome following cyst rupture has not been previously reported.

Because hepatic synthetic function is often preserved, patients with isolated PLD typically have low Model for End‐Stage Liver Disease (MELD) scores, which historically underestimated true physiologic disease severity and limited transplant access. Updated Organ Procurement and Transplantation Network (OPTN) guidance in 2022 expanded MELD exception eligibility for PLD to include measures of functional and nutritional deterioration. We present a patient with symptomatic isolated PLD who progressed from being considered too well for transplantation to developing functional decline, weight loss, and sarcopenia, leading to repeat transplant evaluation, wait‐list registration, and MELD exception approval. Two weeks after listing, he sustained fatal traumatic hepatic cyst rupture with hemoperitoneum and abdominal compartment syndrome.

## 2. Case Presentation

A 39‐year‐old man presented for repeat liver transplant evaluation because of progressive sarcopenia, unintentional weight loss, and functional decline in the setting of symptomatic isolated PLD. He had initially been referred to hepatology in August 2022 after developing several months of abdominal discomfort and a pressure‐like abdominal sensation. His medical history included multiple biopsy‐confirmed hepatic hemangiomas and remote intravenous drug use. He had abstained from alcohol and tobacco for 6 years. His father had died from hepatitis C–associated hepatocellular carcinoma.

At his initial hepatology evaluation, liver enzymes were within normal limits, and cross‐sectional abdominal imaging demonstrated numerous lesions throughout the liver that were characterized as hepatic cysts versus hemangiomas. He underwent an initial transplant assessment, but the evaluation was closed because his low MELD score and overall clinical condition did not support liver transplantation at that time. Longitudinal hepatology follow‐up at approximately 6‐month intervals was recommended. However, because he lived in a rural, out‐of‐state community, follow‐up was inconsistent, and definitive characterization of the hepatic lesions was not completed during that interval.

He subsequently returned for reevaluation after developing a 30‐lb unintentional weight loss over the preceding year, progressive loss of muscle mass, worsening abdominal distention, early satiety, anorexia, and declining functional status. On examination, he had no jaundice or scleral icterus. His abdomen was soft and nontender but notable for pronounced hepatomegaly without ascites or peripheral edema. Laboratory evaluation showed a creatinine level of 0.85 mg/dL, total bilirubin level of 1.7 mg/dL, international normalized ratio of 1.7, sodium level of 138 mEq/L, albumin level of 4.9 g/dL, hemoglobin level of 12.7 g/dL, and platelet count of 131 K/μL, yielding a MELD 3.0 score of 13.

**FIGURE 1:**
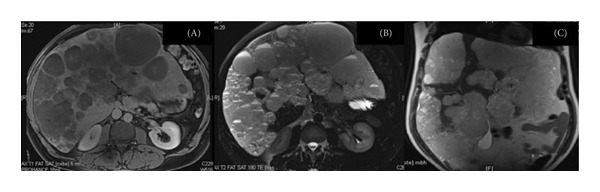
Magnetic resonance imaging (MRI) of the abdomen demonstrating massive hepatomegaly measuring 31 cm (previously 27 cm) with diffuse cystic involvement. (A) Axial T1‐weighted image demonstrating innumerable predominantly hypointense hepatic cysts, several with intrinsic T1 hyperintensity consistent with hemorrhagic or proteinaceous content. (B) Axial T2‐weighted image demonstrating markedly hyperintense cystic lesions with fluid–fluid levels and internal complexity. (C) Coronal T2‐weighted image demonstrating severe craniocaudal hepatomegaly with diffuse cystic replacement, a dominant left hepatic lobe cyst measuring 9.5 × 8.7 cm, and associated narrowing of the left portal vein, distortion of the left and middle hepatic veins, and mild inferior vena cava compression.

Magnetic resonance imaging of the abdomen demonstrated massive hepatomegaly with innumerable hepatic cystic lesions exhibiting fluid–fluid levels. There was severe narrowing of the left portal vein and compression of the middle and left hepatic veins. The pancreas and kidneys appeared normal, without evidence of polycystic kidney disease (Figure 1).

A diagnosis of isolated PLD was established using Reynolds criteria, supported by the absence of a family history of polycystic disease, the absence of renal cysts, and the diffuse hepatic cyst burden. Given the extensive involvement of the liver, surgical options such as fenestration or segmental resection were not feasible. The patient’s progressive symptoms, weight loss, sarcopenia, and functional decline prompted reevaluation for liver transplantation in March 2024. Following multidisciplinary transplant evaluation in April 2024, he was approved for transplantation by the selection committee. Wait‐list registration was deferred pending completion of a required stress echocardiogram, after which he was listed for liver transplantation in May 2024. A MELD exception request was subsequently granted under the 2022 OPTN criteria for symptomatic PLD.

Two weeks later, the patient was admitted following a ground‐level fall and was found to have traumatic rupture of multiple hepatic cysts with hemoperitoneum. His clinical course was complicated by abdominal compartment syndrome and hemorrhagic shock. Interventional radiology attempted arterial embolization; however, despite intensive resuscitative efforts, he ultimately died.

## 3. Discussion

Although many patients with isolated PLD remain asymptomatic with preserved synthetic function, advanced disease may result in significant morbidity from massive hepatomegaly, early satiety, malnutrition, portal hypertension, and cyst‐related complications such as hemorrhage, rupture, of infection [1]. In patients with diffuse cystic involvement, surgical therapies such as fenestration or segmental resection are often not feasible, leaving liver transplantation as the only definitive treatment for symptom relief and improvement in quality of life [6, 7].

The MELD score—originally developed to predict 3‐month mortality in patients undergoing transjugular intrahepatic portosystemic shunt—now serves as the central tool used by the United Network for Organ Sharing (UNOS) for organ allocation [8, 9]. Because MELD relies on laboratory markers of hepatic and renal dysfunction, it accurately predicts mortality in cirrhosis characterized by impaired synthetic function. However, in conditions such as isolated PLD, where morbidity arises from mass‐effect physiology, MELD may substantially underestimate clinical severity. Consequently, patients may experience profound functional decline while maintaining low priority for organ allocation [10].

To address these limitations, MELD exception pathways were developed for diseases in which MELD does not accurately reflect clinical urgency. While hepatocellular carcinoma remains the most common indication for exception, conditions such as cholangiocarcinoma, hepatopulmonary syndrome, and PLD may also qualify [11]. Prior OPTN guidance, however, restricted PLD‐related exceptions to patients with severe symptoms accompanied by renal dysfunction or decompensated liver disease, criteria that excluded many patients whose morbidity stemmed from mass‐effect physiology rather than impaired synthetic function.

In 2022, OPTN updated the PLD exception criteria to better capture disease severity by incorporating clinically meaningful features such as severe complications of portal hypertension, moderate‐to‐severe protein‐calorie malnutrition, and radiographic or clinically confirmed sarcopenia [12, 13]. These revisions better account for disease manifestations that are not represented by laboratory‐based MELD variables. In the present case, the exception pathway functioned as intended. The patient’s progressive weight loss, sarcopenia, functional decline, and severe mass‐effect symptoms were recognized during repeat transplant evaluation, and he was approved for transplantation despite a calculated MELD 3.0 score of 13.

This case also illustrates that transplant candidacy in symptomatic PLD may change substantially over time. When the patient was initially evaluated in 2022, his low MELD score and overall clinical condition did not qualify him for transplantation. However, continued disease progression subsequently led to worsening weight loss, sarcopenia, anorexia, abdominal discomfort, and declining functional status, prompting reevaluation. Thus, an initial determination that a patient is too well for transplantation at that time should not preclude continued surveillance and reassessment when symptom burden evolves.

Importantly, longitudinal reassessment is essential for patients with symptomatic PLD who are initially considered too well for transplantation because nutritional, functional, and mass‐effect complications may progress over time. In this patient, rural out‐of‐state residence and inconsistent follow‐up may have reduced opportunities for serial assessment and timely reevaluation, although the available record cannot establish that these factors directly altered his clinical course. Accordingly, awareness of signs of progressive disease burden despite relatively preserved laboratory findings, as well as familiarity with indications for MELD exception, is important among primary care clinicians and other providers caring for patients with PLD. Greater recognition of these clinical changes and of the MELD exception pathway may facilitate timely referral or repeat transplant evaluation.

After his worsening nutritional and functional status was recognized, the patient underwent transplant assessment, was approved by the selection committee, and was listed after completion of his stress echocardiography. He sustained traumatic hepatic cyst rupture only 2 weeks after placement on the transplant waiting list. This interval does not indicate an excessive wait for organ allocation or failure of the MELD exception pathway. Rather, it illustrates the ongoing and unpredictable vulnerability of patients with advanced symptomatic PLD while awaiting transplantation.

A distinctive feature of this case is the development of traumatic hepatic cyst rupture with massive hemoperitoneum, an event reported only rarely in the literature [5]. Although cyst infection and spontaneous hemorrhage are recognized complications, traumatic cyst rupture leading to hemorrhagic shock is exceedingly rare [14, 15]. To our knowledge, the development of abdominal compartment syndrome following these complications has not been previously reported. This case demonstrates the potential for minor trauma to precipitate catastrophic hemorrhagic complications in patients with severe cyst burden. Clinicians should consider cyst rupture or hemorrhage in patients with known PLD who present with a change in abdominal pain, distention, anemia, or hemodynamic instability following trauma.

## 4. Conclusion

Taken together, this case illustrates the dynamic progression of symptomatic isolated PLD and the limitations of laboratory‐based measures in capturing nutritional, functional, and mass‐effect disease burden. Although the patient was initially considered too well for transplantation, subsequent sarcopenia, weight loss, and functional decline appropriately prompted repeat evaluation, wait‐list registration, and MELD exception consideration. His fatal traumatic cyst rupture shortly after listing was rare and unpredictable and does not indicate failure of the exception pathway. Rather, this case underscores the importance of longitudinal surveillance and timely reassessment when patients with PLD develop progressive nutritional or functional deterioration despite relatively preserved laboratory findings.

## Author Contributions

Zachary C. Vinton: reviewed patient records, reviewed literature, was responsible for the overall design, and drafted the manuscript.

Carter A. Schulz: participated in design of the manuscript, contributed to writing of initial drafts, and extensive revisions.

Stephanie J. Melquist: participated in design of the manuscript, contributed to writing of initial drafts, provided substantial editing of the manuscript, and mentored the project.

Clark C. Kulig: supervised the creation of the manuscript, provided final revisions, and obtained patient consent.

## Funding

No funding was received for this work.

## Ethics Statement

This study was determined to be exempt from institutional review board oversight in accordance with institutional policy.

## Consent

The patient provided verbal informed consent for publication prior to death. Written consent could not be obtained due to the patient’s death and inability to contact next of kin despite exhaustive efforts. The case has been fully anonymized.

## Conflicts of Interest

The authors declare no conflicts of interest.

## Data Availability

The data that support the findings of this study are available on request from the corresponding author. The data are not publicly available due to privacy or ethical restrictions.
